# Late-Onset Recurrent Femoral Cyst Formation 10 Years after ACL Reconstruction: A Case Report and Literature Review

**DOI:** 10.1155/2020/3586981

**Published:** 2020-03-23

**Authors:** Enrique Sanchez-Munoz, Antonio Maestro Fernández, Iván Pipa Muñiz, Nicolas Rodríguez García

**Affiliations:** ^1^Knee Unit, Department of Orthopaedic Surgery and Traumatology, University Hospital of Toledo, Avenida Barber 30, ZIP 45004 Toledo, Spain; ^2^Knee Pathology and Sports Medicine Unit, HM IMI Clinic, Av. Irlanda, 21, 45005 Toledo, Spain; ^3^Real Sporting de Gijón SAD, Department of Orthopaedic Surgery, Begoña Hospital, Av. Pablo Iglesias, 92, 33204 Gijón, Asturias., Spain; ^4^Department of Orthopaedic Surgery, Begoña Hospital, Av. Pablo Iglesias, 92, 33204 Gijón, Asturias., Spain; ^5^Department of Orthopaedic Surgery and Traumatology, Hospital de la Cruz Roja, Calle Uría, 37, ZIP 33202 Gijón, Asturias., Spain

## Abstract

Synovial fistula and cyst formation after anterior cruciate ligament (ACL) reconstruction is very unusual and almost always affects the tibia. Only 3 cases originating in the femur have been reported. We present a rare case of late-onset femoral cyst formation related to ACL reconstruction. Ten years after successful ACL reconstruction surgery, effusion and pain at the lateral aspect of the lateral femoral condyle appeared. Symptoms persisted despite initial percutaneous drainage and conservative treatment. Surgery was carried out, with drainage and graft-fixation pin device removal, with recurrent cyst formation after the intervention. Total recovery of the patient was achieved after carrying out revision surgery consisting in bone tunnel filling using autologous bone graft and occlusion of cortical bone defect with local fascial flap. The development of this unusual complication was related to lack of absorption of the fixation device, bone burn due to drilling, and persistent synovial fluid in the femoral bone tunnel, combined with the absence of bone ingrowth.

## 1. Introduction

Anterior cruciate ligament (ACL) lesions are among the most common sports-related knee injuries, with a growing incidence [1], thus making ACL reconstruction surgery a common orthopaedic procedure [1]. Despite the high number of ACL reconstruction surgeries, complications related to fixation system devices are unusual. In particular, synovial fluid fistula and cyst formation is reported to have a very low incidence that ranges between 0.61% and 0.011% [2–5]. Between synovial fistula and cyst, femoral cyst formation is especially unusual, with only three cases described to date [5–7]. The current report presents a case of femoral cyst formation after ACL reconstruction. This is an exceptional case not only for being the fourth one reported, but also due to some unique features: late onset ten years after surgery and symptoms persisted despite loosened hardware removal. Due to this, a second revision surgery was required in which bone marrow defect was filled with autologous bone graft and cortical bone defect was covered with local fascial flap.

## 2. Case Report

A 45-year-old male patient presented with persistent pain and effusion at the lateral aspect of his right knee. He underwent an uneventful ACL reconstruction ten years before, with total functional recovery and no instability or pain symptoms after full rehabilitation postoperative program. For ACL graft fixation, a bioabsorbable crosspin was used at the femur and a bioabsorbable interference screw was used at the tibia. The patient reported that he did not suffer any trauma, increased sports activity level, or other remarkable events in the weeks or months prior to the moment when pain and effusion started. The patient was first treated at another centre where femoral crosspin loosening was noted. After a magnetic resonance image (MRI) showed an intact ACL graft and no other complications, the patient underwent surgery for the removal of a femoral crosspin.

**Figure 1:**
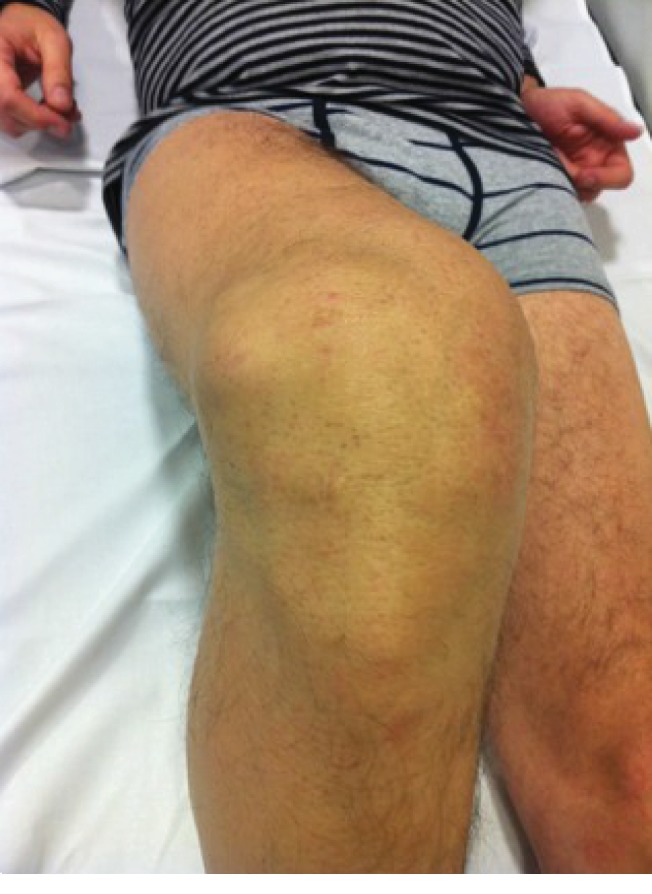
Swelling and cyst formation at the lateral aspect of the knee ten years after a noncomplicated ACL reconstruction. The cyst is underneath the skin incision that was made for the introduction of the crosspin device for femoral graft fixation.

One month after femoral crosspin removal surgery, effusion and pain persisted. Then, the patient was evaluated at our hospital for the first time. Physical exploration showed swelling at the lateral aspect of the lateral femoral condyle (Figure 1). A rounded, cyst-like mass with well-defined borders was evident at palpation. No local signs of infection were found. Lachman, pivot-shift, and anterior and posterior drawer tests were negative, with a full range of motion and no valgus or varus instability at physical exploration. Plain anteroposterior and lateral radiographs of the knee showed adequate tunnel position and no tunnel widening.

Suspected diagnosis was postsurgical seroma. Treatment included percutaneous drainage, nonsteroidal anti-inflammatory drugs (NSAIDs), and RICE (rest, ice, compression, and elevation). Samples from the drained seroma were sent for microbiological analysis and culture, with no bacterial ingrowth. Prophylactic antibiotic treatment was prescribed until a negative result of synovial fluid cultures was obtained. Initial evolution was satisfactory, with complete symptom release for 6 months. After this period, recurrent swelling at the lateral aspect of the knee reappeared and remained despite percutaneous drainage. A cyst-like rounded mass was palpable at the same location of previous episodes. Physical exploration was similar to our first evaluation; no local or systemic infection symptoms were found.

**Figure 2:**
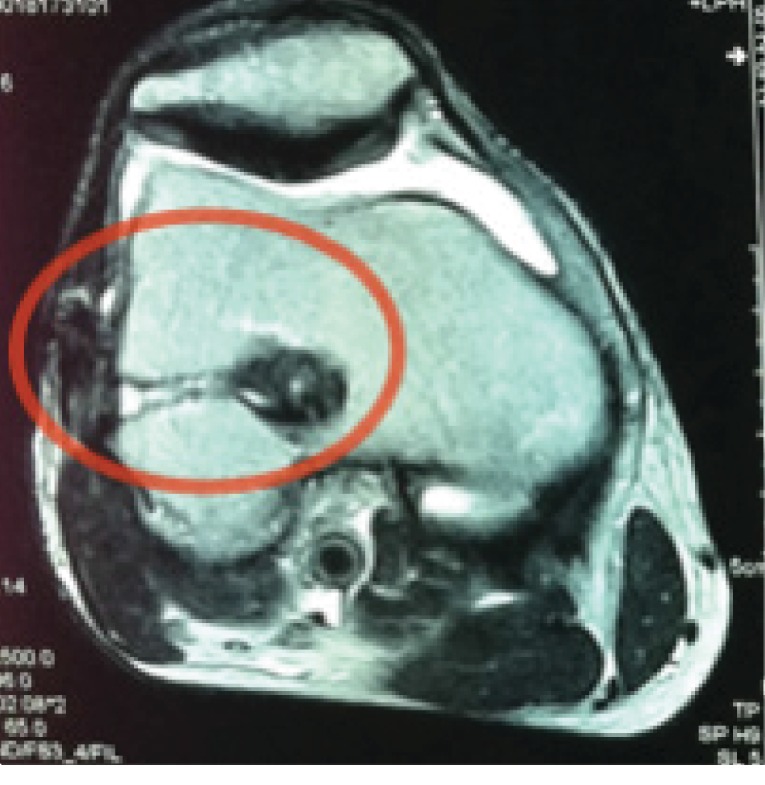
Magnetic resonance image (MRI) showing the fistula, marked with a red circle, that connects the ACL graft femoral tunnel to the cortical bone. The tunnel is filled with synovial fluid which comes from inside the joint. This image also shows the soft tissue disruption in the lateral aspect of the femoral condyle.

MRI showed a cyst formation at the lateral aspect of the knee, with fluid signal in the graft femoral tunnel and around the crosspin tunnel entrance at the lateral cortex of the lateral femoral condyle (Figure 2).

**Figure 3:**
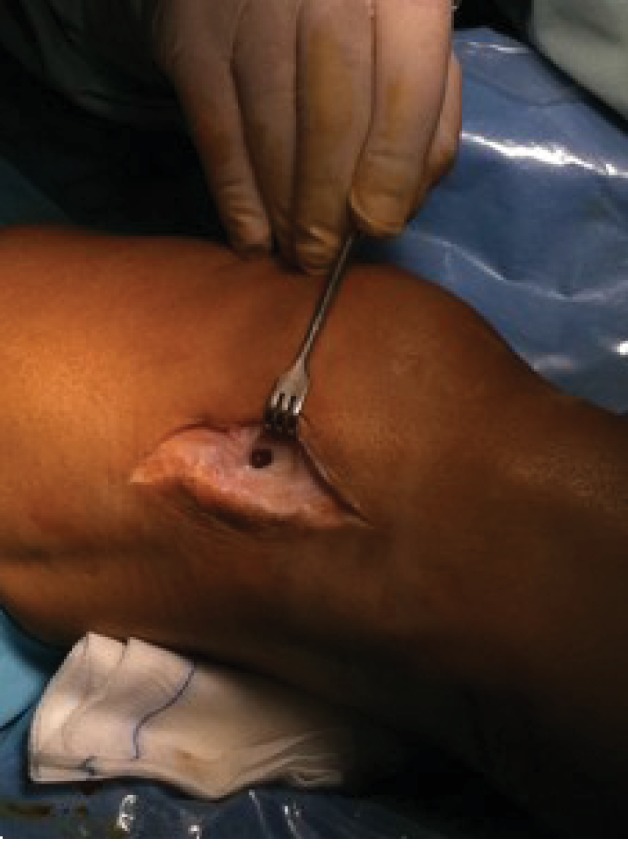
Intraoperative picture showing the cortical bone defect that originates the cyst at the lateral aspect of the lateral femoral condyle.

Surgical treatment in collaboration with plastic surgery was carried out. With the patient in supine position and under ischaemia of the leg, standard knee arthroscopy was carried out. Full knee arthroscopic exploration showed an intact ACL graft with no meniscal or articular cartilage lesions. No widening of the femoral or tibial ACL graft tunnels was noted, and neither was there any graft loosening. During arthroscopy, as a result of fluid inflow to the knee, the lateral cyst increased in size, making it more perceptible at the lateral aspect of the knee. This finding confirmed that a fistula was connecting the knee joint to the lateral condyle; as noted in the previous MRI, this fistula was the origin of the cyst. No signs of septic arthritis were found. After knee arthroscopy, the operation proceeded with open surgery through a lateral approach using the previous surgical scarf from the crosspin removal surgery. The lateral cortex of the lateral femoral condyle was exposed. The hole at the cortical bone of the lateral aspect of the lateral femoral condyle, caused by the drilling carried out to introduce the crosspin used for femoral ACL graft fixation, persisted (Figure 3) with rounded soft tissue burn. At the lateral aspect of the femoral condyle, a cyst-like cavity was found. Once opened, it was filled with synovial fluid and no capsule or synovial tissue surrounded the cyst; however, it has dissected a cavity between bone cortical and deep muscular fascia. No signs of local infection were present. At the lateral end of the femoral tunnel, where the tunnel from the removed crosspin persisted, a burned bone was also noted. The bone tunnel end was surrounded with devitalized bone with negative paprika sign and sclerotic edges; bone edges and surrounding soft tissues were darkened due to burning during drilling. Owing to a small tunnel width of 10 mm, arthroscopic exploration introducing an arthroscope through the tunnel was not possible. The cyst was excised, and burned bone was refreshed until a bleeding marrow bone bed was obtained.

The bone tunnel was filled with autologous marrow bone graft before covering the cortical bone defect with local fascial flap. Postoperative course was uneventful and total recovery was achieved.

Three years after the femoral tunnel revision surgery, the patient remained with full functional recovery and without any clinical symptoms, effusion, or pain recurrence.

## 3. Discussion

ACL reconstruction is a standard and common procedure, with growing incidence [1] and low morbidity rates [2]. Synovial fistula is an uncommon complication (0.61%-0.011% incidence) [2–4]. It usually appears through arthroscopy portals, either chronic [5, 8] or acute [9]. This complication could present as a fistula if opened to skin [4, 9, 10] or as a cyst if skin is preserved [5–7, 9, 11–13]. Usual locations comprise arthroscopy portals and skin incisions for graft introduction and fixation [4–7, 9–13]. Identified risk factors are persistent synovial fluid drainage through tibial bone tunnels [7, 11], size mismatch of interference screws and bone tunnels [12], breakage or absorption of interference screws [8, 13], inadequate or eccentric bone graft placement [14], graft necrosis [11], interference screw loosening [8], and foreign body reaction [15]. Neither ACL graft failure nor infection had been associated with this complication [1, 4, 8, 11].

To our best knowledge, only three cases of femoral cyst formation have been described to date [5–7]. In the study of Feldmann and Fanelli [6], it occurred eight months after surgery, with cyst formation due to foreign body reaction related to nonabsorbable sutures used as passing sutures for tunnel graft placement. Sharma et al. [7] reported fistula formation as a result of extra-articular, extraosseous migration of a femoral interference screw, which is similar to our case, except that our patient had a crosspin instead of an interference screw. The case reported by Faunø et al. [5] is also quite similar to ours, with late onset, four years after ACL reconstruction. However, two features make our case unique within the current published literature: late presentation—10 years after ACL reconstruction surgery—and persistence of symptoms despite the removal of the loosened fixation device (transfixing crosspin). In this patient, a second revision surgery was necessary, with bone tunnel defect filling and soft tissue reconstruction with local flap for the coverage of the cortical bone defect.

Cyst and fistula formation after knee arthroscopy is rather uncommon; usually, several factors are involved in its development. Some of these factors are crucial features that require further study to avoid this complication. Adequate interference screw and bone tunnel match, careful soft tissue closure, and avoidance of bone necrosis related to drilling [7] should always be considered. A deeper understanding of bioabsorbable screw degradation and absorption [16, 17] and its relationship with loosening and osteolysis [8, 16, 17] is the goal for future research.
